# Body Composition Symmetry in Long-Term Active Middle-Aged and Older Individuals

**DOI:** 10.3390/ijerph18115956

**Published:** 2021-06-02

**Authors:** Silvia Stagi, Alessia Moroni, Margherita Micheletti Cremasco, Elisabetta Marini

**Affiliations:** 1 Department of Life and Environmental Sciences, Neuroscience and Anthropology Section, University of Cagliari, Cittadella di Monserrato, 09042 Cagliari, Italy; 2 Department of Life Sciences and Systems Biology, University of Torino, 10123 Torino, Italy; alessia.moroni@unito.it (A.M.); margherita.micheletti@unito.it (M.M.C.)

**Keywords:** body symmetry, segmental body composition, bioelectrical impedance vector analysis, BIVA

## Abstract

This study aimed to analyze body composition and strength symmetry in a sample of 165 middle-aged and elderly Italian volunteers, which included 97 active (67 men and 30 women; 61.17 ± 7.56 years) individuals regularly engaged in Tai Chi Chuan, tennis, or running, and a control group of 59 age-matched sedentary (27 men and 32 women) individuals. Anthropometric and bioelectrical measurements and hand grip strength of both sides were collected. Segmental body composition was analyzed through *specific* bioelectrical impedance vector analysis. The body composition of the right and left limbs was similar among active individuals (arms: T^2^ = 6.3, n.s.; legs: T^2^ = 5.0, n.s.), with a similar pattern in the three different disciplines. By contrast, the control group showed bilateral asymmetry (arms: T^2^ = 6.8, *p* < 0.001; legs: T^2^ = 8.8, *p* < 0.001), mainly because of the higher values of *specific* reactance (t = 2.4; *p* = 0.018) and phase angle (t = 2.0; *p* = 0.054) in the dominant arm, and the higher *specific* vector length (t = −3.0; *p* = 0.027) in the left leg. All of the groups showed a higher hand grip strength in the dominant arm (active: t = 7.0, *p* < 0.001; control: t = 2.9; *p* < 0.01). In conclusion, the active individuals showed stronger body composition symmetry than the controls, thus indicating a previously undetected positive effect of sport in middle-aged and older adults.

## 1. Introduction

The ageing process exposes the older population to the risk of malnutrition, sarcopenia, sarcopenic obesity, and frailty. These conditions can significantly accelerate functional decline and increase the risk of morbidity and falls, which in turn are related to a greater risk of mortality [1]. Such a scenario is exacerbated when combined with psychological disorders, physical inactivity, or poor dietary habits.

Physical activity (PA) can effectively contribute to the maintenance of well-being in the elderly, thus representing a driving force for successful ageing. Indeed, PA helps counter the age-related trend toward a decline of muscle mass and functionality, and an increase and central accumulation of fat mass (FM) [2,3]. Accordingly, the World Health Organization recommends PA and muscle strengthening training for maintaining physical, psychological, and cognitive well-being in older-aged individuals [4]. Older people should also exercise to improve their balance and prevent falls in daily living.

Body asymmetry, specifically, strength, functionality, and body composition asymmetry, could interfere with physical balance and risk of falls. Sports science highlights the relevance of maintaining body symmetry in order to improve technical proficiency performance and to prevent injuries [5]. In the older population, the literature shows a positive correlation between asymmetry and functional disabilities, body balance, and falls [6,7,8,9,10]. However, research in the older population mainly refers to strength and functional asymmetry, while the role of body composition asymmetry has been poorly investigated. Moreover, to the best of our knowledge, only a few studies have investigated the effects of PA on body composition asymmetry in older individuals, and participants were limited to tennis players [11,12].

Considering the gap in the literature, this study focused on the analysis of strength and body composition asymmetry in a sample of long-term active older individuals engaged in Tai Chi Chuan, tennis, or running.

## 2. Methods

The study sample included 97 middle-aged and older individuals (67 men and 30 women; 61.17 ± 7.56 years) involved in Tai Chi Chuan (33 individuals; 6 years of practice on average), tennis (29 individuals; 22 years on average), or running (35 individuals 17 years on average). These modalities were selected because of their practicability until old age.

The control group consisted of 59 individuals (27 men and 32 women; 61.96 ± 8.18 years) living in the same geographical area and not performing regular physical exercise, but doing normal everyday activities (including manual labor).

Exclusion criteria were the presence of physical handicaps, pathologies that might influence the measurements (e.g., significant cardiovascular or pulmonary diseases, endocrine or renal diseases, cancer, or severe inflammatory conditions), metallic prostheses, pacemakers, or limb amputations.

Participant recruitment was performed during the period from January 2018 to April 2019. For each subject, anthropometric and bioimpedance measurements were taken on the same day, beginning with the anthropometric ones.

This study was approved by the Independent Ethical Committee of the A.O.U. of Cagliari (PG/2017/1700). Each participant was informed about the purposes and methods of the study, and signed the informed consent form in order to participate.

### 2.1. Anthropometric Measurements and Hand Grip Strength

Anthropometric measurements were performed by an ISAK-certified anthropometrist, in agreement with international standards [13].

Height (cm) and weight (kg) were taken using a stadiometer (SECA, Hamburg, Germany) and a mechanical scale (SECA, Hamburg, Germany), respectively. Body mass index (BMI) was calculated using the formula weight/height^2^ (kg/m^2^). The circumferences (C, cm) of the mid-arm and calf, and lengths (L, cm) of the arm (distance between the acromion and the stylion) and leg (distance between the great trochanter and the malleolus) were measured on both sides of the body using a body tape (SECA, Hamburg, Germany).

Hand grip strength was measured using a hydraulic dynamometer (Sahean Corporation, MSD, Brussels, Belgium). The participant was asked to stand upright and hold the instrument and squeeze it with the greatest possible force, with his/her elbow flexed at 90° [14].

The measurement was carried out three times for each side, alternately, in order to guarantee a few seconds of recovery. The maximum value obtained from the three repetitions was used for the analysis.

### 2.2. Bioelectrical Measurements

Bioelectrical impedance measurements were carried out using BIA 101 single-frequency devices (Akern BIA 101 and Akern BIA 101 New Edition, Akern Srl, Firenze, Italy) and Biatrodes electrodes (Akern Srl, Firenze, Italy).

Before each session, the device was checked with a calibrated circuit, whose impedance values are as follows: R = 380 Ω, X_c_ = 47 Ω (±2% error). The measurements were taken in the morning and participants were asked not to drink or eat, and to empty their bladders, wear light clothing, and remove metal objects before the examination.

To assess the body composition of the dominant and non-dominant arms, and the right and left legs, segmental *specific* bioelectrical impedance vector analysis (BIVA), an accurate procedure recently tested for the evaluation of the segmental body composition, was applied [15]. Resistance (R) and reactance (X_c_) values for each body segment were adjusted for a correction factor (A/L), where A (cm^2^) is the cross-sectional area (C^2^/4π cm^2^) of the mid-arm and calf, and the L (cm) is the length of the arm and leg.

*Specific* impedance (Z_sp_) was calculated as (R_sp_^2^ + X_csp_^2^)^0.5^ (Ω cm). The phase angle (PhA) was obtained using the formula arctan X_c_/R 180/π (degree).

According to the *specific* BIVA, the vector length is positively related to variations of FM percentage (%FM), while PhA is positively related to body cell mass, muscle mass in particular, and to the intracellular/extra cellular water (ICW/ECW) ratio.

### 2.3. Statistical Analysis

The bias between the bioelectrical values obtained with different devices was amended using a correction factor calculated ad hoc [16], and applied to raw data obtained with the Akern BIA 101 New Edition (arm: R = +0.65, X_c_ = +4.97; leg: R = −0.35, X_c_ = +4.48).

Descriptive statistics were calculated for all of the variables and for each sex separately.

Differences in age and anthropometric characters between the active and control groups, considering sex, were analyzed using two-way ANOVA.

Differences between the limbs of the two sides (dominant vs. non-dominant arm, and right vs. left leg) were analyzed using paired Student’s t test, considering Cohen’s d effect size and Hotelling’s T^2^ test, and were graphically represented in the paired data RX_c_ graphs. In these graphs, ellipses overlapping the origin indicated no differences between the bioelectrical values of the two sides. By contrast, non-centered ellipses indicated significant differences between sides, with the effect of R or X_c_ prevailing when the ellipse approached the corresponding axis. The level of asymmetry was compared between limbs, age subgroups (<60 and ≥60 years), and modalities by means of the Hotelling’s T^2^ test. 

Statistical analyses were performed using SPSS version 25 (SPSS Inc. Chicago, IL, USA), classic BIVA [17] (and *specific* BIVA (www.specificbiva.com, accessed on 31 May 2021) software.

## 3. Results

The active participants of both sexes exhibited similar age, higher stature, and lower weight and BMI than the controls (Table 1). Both groups showed a normal pattern of sexual dimorphism, with a higher stature, weight, and BMI among men (Table 1).

Descriptive and comparative statistics of the bioelectrical values in the dominant and non-dominant arms, and the right and left legs, along with the hand grip strength are shown in Table 2.

**Figure 1:**
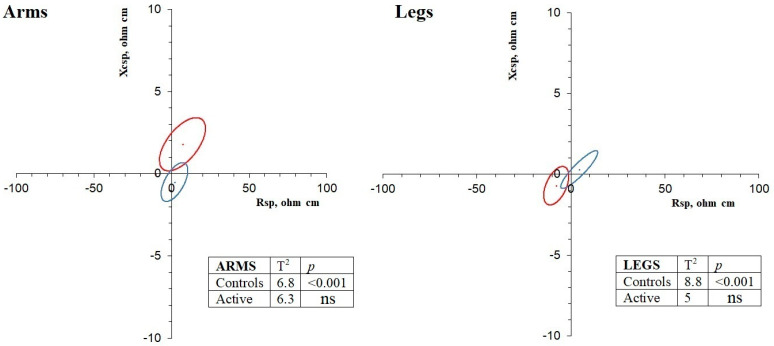
Paired data RX_c_ graphs and results of the Hotelling’s T^2^ test for differences between contralateral limbs. Blue: active subjects; Bordeaux: controls.

**Figure 2:**
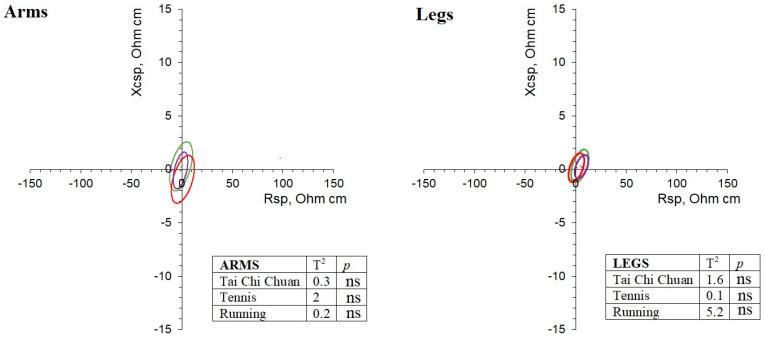
Paired data RX_c_ graphs and results of the Hotelling’s T^2^ test for differences between the contralateral limbs. Green: Tai Chi Chuan; red: tennis; purple: running.

Active participants showed limb symmetry, as indicated by the paired Hotelling’s T^2^ test and the overlap of the ellipses with the origin of the graph (Figure 1), and as confirmed by the paired Student’s t test, which showed no differences between the bioelectrical values of the two sides (Table 2). The only exceptions were the univariate comparisons of Rsp and Zsp (Table 2), which indicated a slightly higher %FM in the right leg. The symmetrical pattern was similar between the limbs and between age subgroups (<60 and ≥60 years). The symmetrical body composition among athletes was also observed considering the different disciplines separately. The paired confidence ellipses of the Tai Chi Chuan, tennis, and running groups were all centered on the origin of the graph, and the bivariate comparison between sides was always not significant (Figure 2), as well as the comparison of asymmetry levels among disciplines. The univariate comparisons also indicated the lack of significant differences between sides for the arms in all of the groups, with the exception of a tendency to higher Rsp values (higher %FM) in the right legs of runners (*p* = 0.034).

By contrast, the control group showed body composition asymmetry, as indicated by the significant paired Hotelling’s T^2^ test results and the position of the ellipses in the graph that at no point overlapped the center (Figure 1). Asymmetry was due to the higher values of X_csp_ and PhA in the dominant arm and to the higher values of R_sp_ and Z_sp_ in the left leg, as indicated by the position of the ellipses and univariate comparisons (Table 2). The legs were significantly more asymmetrical than the arms (T^2^ = 9.6, *p* = 0.010). Age subgroups showed similar levels of asymmetry in both the arms and legs.

Among all of the active and sedentary participants, hand grip strength was significantly higher in the dominant hand (Table 2).

## 4. Discussion

The human body exhibits laterality and the differential use of two limbs can determine asymmetry in body composition and strength. Such features can be influenced by PA, can vary in the life course, and have been shown to be related to injuries in athletes [18,19,20] and to health outcomes in older people [6,7,8,9,10].

In this study, active individuals showed bilateral symmetry in body composition, with a similar pattern in the upper and lower limbs, in the two age subgroups and in the three disciplines, despite their different training modalities and physical effects. By contrast, the control subjects, regardless of their age, were characterized by limb asymmetry, with higher muscle mass (as indicated by the higher X_csp_ and PhA values) in the dominant arm and higher values of %FM (higher R_sp_ and Z_sp_) in the left leg.

In both the control and active groups, the dominant arm showed higher hand grip strength than the opposite one.

Studies on body composition asymmetry in the general population, including older people, show that the dominant leg and arm are characterized by higher values of lean mass [5,21] and less accentuated differences in the FM [5].

The effect of sports on body asymmetry in middle-aged and older adults has mostly been investigated in tennis players, and the results of previous studies are inconsistent. In line with the present study, Piasecki et al. [12] detected symmetrical muscle size in the arms of men. By contrast, Ireland et al. [11] observed greater muscle and bone size in the racquet arm.

Differences in strength between dominant and non-dominant sides have been frequently observed in healthy adults [22,23,24]. The asymmetry of hand grip strength in healthy older subjects shows a less homogeneous pattern than that in the general population, and in some studies, a symmetrical strength was observed [6,8,25]. Studies on active older participants focused on tennis players consistently detected a greater strength in the dominant arm [11,12,26,27], as in this study.

Strength asymmetry has been related to health outcomes in older people, particularly to functional disabilities, body balance, and falls [7,8,9,10], especially among pre sarcopenic and sarcopenic individuals [10].

The role of body composition asymmetry on health is less defined. According to recent studies, body composition symmetry of the lower limbs has a lesser effect on health [21] than strength symmetry [10,21] or good levels of muscle mass in both limbs [28], which could be related to postural control [29].

The results of this study suggest that PA can induce body composition symmetry, likely due to exercise that involves, in some degree, all limbs. By contrast, the age-related reduction of PA characterizing the general population could favor differential use of the contralateral limbs in daily and work-related activities in favor of the dominant side.

The effect of PA on hand-grip strength asymmetry appears to be less pronounced.

More studies are needed to better define the variability of the age-related trend of body symmetry among older individuals, when the physiological trend of reducing muscle mass and strength becomes predominant.

This study has some limitations that should be listed. First, the results are not generalizable to other populations or individuals with clinical conditions and are not directly comparable with studies realized using other bioimpedance devices. Second, the influence of socio-cultural factors, diet, lifestyle, and physical fitness components has not been analyzed. However, to the best of our knowledge, this study is one of the very few studies on body composition asymmetry in aged long-term active individuals of both sexes.

## 5. Conclusions

The results of this study showed that, along with the general positive effect on nutritional status, PA appears to play a role in maintaining the symmetry of body composition, thus indicating a possible positive effect of sport that was not previously detected.

## Figures and Tables

**Table 1:** Descriptive statistics of the control and active individuals and two-way ANOVA for comparisons of the groups.

	Control		Active		ANOVA		
	Men (27)	Women (32)	Men (67)	Women (30)			
	Mean SD	Mean SD	Mean SD	Mean SD	*p*_sex_	*p*_sport_	*p*_sex·sport_
Age	62.13 ± 8.53	61.66 ± 7.75	61.24 ± 7.54	60.84 ± 7.47	0.741	0.520	0.979
Height	167.92 ± 6.37	154.28 ± 7.25	170.56 ± 6.93	156.70 ± 6.27	0.000	0.032	0.925
Weight	77.61 ± 10.39	63.56 ± 9.48	70.92 ± 9.19	53.39 ± 7.21	0.000	0.000	0.270
BMI	27.44 ± 2.60	26.77 ± 4.01	24.37 ± 2.71	21.75 ± 2.71	0.002	0.000	0.061

BMI: body mass index.

**Table 2:** Descriptive and comparative statistics of the bioelectrical values and hand grip strength.

	**Controls**				**Paired *t*-Test**		**Effect Size**	**Active**				**Paired *t*-Test**		**Effect Size**
	**Men (27)**		**Women (32)**					**Men (67)**		**Women (30)**				
**ARMS**	**D**	**ND**	**D**	**ND**				**D**	**ND**	**D**	**ND**			
	**Mean SD**	**Mean SD**	**Mean SD**	**Mean SD**	***t***	***p***	***d***	**Mean SD**	**Mean SD**	**Mean SD**	**Mean SD**	***t***	***p***	***d***
R_sp_	266.3 ± 44.5	264.8 ± 40.78	330.7 ± 62.84	328.1 ± 63.30	0.582	0.563	0.045	231.6 ± 29.28	232.9 ± 32.249	274.4 ± 48.29	284.5 ±52.32	−1.659	0.101	0.176
X_csp_	31.6 ± 7.61	30.1 ± 6.62	31.3 ± 8.13	30.6 ± 8.24	2.438	0.018	0.303	25.8 ± 4.81	25.3 ± 5.317	25.3 ± 5.82	25.5 ± 5.20	0.826	0.411	0.082
Z_sp_	268.3 ± 44.90	266.6 ± 40.96	332.2 ± 63.05	329.6 ± 63.59	0.609	0.545	0.049	233.1 ± 29.43	234.3 ± 32.43	275.6 ± 48.42	285.7 ± 52.43	−1.631	0.106	0.174
PhA	6.7 ± 1.12	6.5 ± 1.16	5.4 ± 1.05	5.3 ± 1.0	1.969	0.054	0.267	6.4 ± 0.92	6.2 ± 0.97	5.3 ± 0.97	5.1 ± 0.79	1.737	0.086	0.185
H.G.	38.1 ± 9.36	36.9 ± 8.49	23.5 ± 5.45	22.6 ± 5.45	2.885	0.005	0.337	40.6 ± 7.67	38.0 ± 7.29	25.3 ± 5.09	22.3 ± 5.72	7.080	0.000	0.697
**LEGS**	**R**	**L**	**R**	**L**				**R**	**L**	**R**	**L**			
	**Mean SD**	**Mean SD**	**Mean SD**	**Mean SD**	***t***	***p***	***d***	**Mean SD**	**Mean SD**	**Mean SD**	**Mean SD**	***t***	***p***	***d***
R_sp_	255.2 ± 44.42	261.2 ± 52.30	297.9 ± 58.52	303.4 ± 53.67	−2.983	0.004	0.265	250.3 ± 23.49	244.9 ± 23.53	280.1 ± 31.12	279.3 ± 35.04	2.243	0.027	0.266
X_csp_	31.3 ± 9.63	30.62 ± 9.11	31.7 ± 9.92	33.4 ± 9.71	−1.582	0.119	0.178	29.5 ± 5.86	29.1 ± 6.06	30.9 ± 7.55	31.1 ± 7.09	0.922	0.359	0.163
Z_sp_	257.1 ± 45.00	263.0 ± 52.82	299.7 ± 58.94	305.2 ± 54.18	−2.979	0.004	0.263	252.1 ± 23.80	246.7 ± 23.86	282.0 ± 30.70	281.2 ± 34.88	2.240	0.027	0.265
PhA	6.9 ± 1.40	6.6 ± 1.20	6.0 ± 1.33	6.2 ± 1.16	0.562	0.577	0.044	6.7 ± 1.01	6.7 ± 1.07	6.4 ± 1.99	6.4 ± 1.70	−0.541	0.590	0.066

R_sp_: *specific* resistance; X_csp_: *specific* reactance; Z_sp_: *specific* vector length; PhA: phase angle; D: dominant; ND: non-dominant; R: right; L: left; H.G.: hand grip strength; *t*: paired Student’s t test; *p*: significance; *d*: Cohen’s d for paired samples.

## Data Availability

The data that support the findings of this study are available from the corresponding author, upon request.
